# Mining the role of angiopoietin‐like protein family in gastric cancer and seeking potential therapeutic targets by integrative bioinformatics analysis

**DOI:** 10.1002/cam4.3100

**Published:** 2020-05-14

**Authors:** Cheng Tang, Erbao Chen, Ke Peng, Haiwei Wang, Xi Cheng, Yan Wang, Shan Yu, Yiyi Yu, Yuehong Cui, Tianshu Liu

**Affiliations:** ^1^ Department of Medical Oncology Zhongshan Hospital Affiliated to Fudan University Shanghai PR China

**Keywords:** angiopoietin‐like proteins, functional enrichment, gastric cancer, prognostic value, transcriptional expression level

## Abstract

**Background:**

The indistinctive effects of antiangiogenesis agents in gastric cancer (GC) can be attributed to multifaceted gene dysregulation associated with angiogenesis. Angiopoietin‐like (ANGPTL) proteins are secreted proteins regulating angiogenesis. They are also involved in inflammation and metabolism. Emerging evidences have revealed their various roles in carcinogenesis and metastasis development. However, the mRNA expression profiles, prognostic values, and biological functions of ANGPTL proteins in GC are still elucidated.

**Methods:**

We compared the transcriptional expression levels of ANGPTL proteins between GC and normal gastric tissues using ONCOMINE and TCGA‐STAD. The prognostic values were evaluated by LinkedOmics and Kaplan–Meier Plotter, while the association of expression levels with clinicopathological features was generated through cBioPortal. We conducted the functional enrichment analysis with Metascape.

**Results:**

The expression of ANGPTL1/3/6 was lower in GC tissues than in normal gastric tissues. High expression of ANGPTL1/2/4 was correlated with short overall survival and post‐progression survival in GC patients. Upregulated ANGPTL1/2 was correlated with higher histological grade, non‐intestinal Lauren classification, and advanced T stage, while ANGPTL4 exhibited high expression in early T stage, M1 stage, and non‐intestinal Lauren classification.

**Conclusions:**

Integrative bioinformatics analysis suggests that ANGPTL1/2/4 may be potential therapeutic targets in GC patients. Among them, ANGPTL2 acts as a GC promoter, while ANGPTL1/4’s role in GC is still uncertain.

## Graphical abstract

Integrative bioinformatics analysis suggests that ANGPTL1/2/4 may be potential therapeutic targets in GC patients. Among them, ANGPTL2 acts as a GC promotor, while ANGPTL1/4’s role in GC is still uncertain.

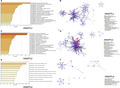

## 1 INTRODUCTION

Gastric cancer (GC) is the fifth most common cancer in the world and the second leading cause among death from cancer. Taking the significant role of angiogenesis during cancer development into consideration, antiangiogenesis agents are expected to improve GC patients’ prognosis significantly. However, so far, only vascular endothelial growth factor receptor (VEGFR) antibody ramucirumab^1^ and VEGFR‐targeted tyrosine kinase inhibitor (TKI) apatinib^2^ have showed slight advantage of survival in the second‐ and third‐line therapy of advanced GC, respectively. This phenomenon can be attributed to multifaceted gene dysregulation and complex molecular mechanisms associated with angiogenesis. Thus, figuring out other novel biomarkers related to angiogenesis process in GC may be helpful to improve the precision and efficacy of therapies.

Angiopoietin‐like (ANGPTL) proteins are a family of secreted glycoproteins structurally similar to the angiopoietins.^3^ To date, eight ANGPTL proteins have been discovered, namely from ANGPTL1 to ANGPTL8. Both angiopoietin and ANGPTL protein family are characterized by two domains: an N‐terminal coiled‐coil domain and a C‐terminal fibrinogen‐like domain.^3^ Although the sequence is similar, ANGPTL proteins do not bind to the same receptors, named Tie2 or Tie1, as angiopoietins do. Instead, some of them bind to other kinds of receptors, such as leukocyte immune‐globulin‐like receptor B2 (LILRB2),^4^ integrins α5β1,^5^ and some of them are orphan ligands.^4^

Angiopoietins/Tie receptor signaling is involved in modulating angiogenesis and preserving vascular integrity and permeability.^6^ Notwithstanding, none of the ANGPTL proteins bind to the angiopoietin receptors, most members still show effects on angiogenesis.^7^ ANGPTL proteins also exhibit many other biological properties in lipid, glucose and energy metabolism, inflammation, hematopoiesis, as well as cancer progression and metastasis.^3^, ^7^, ^8^

ANGPTL proteins are widely expressed in many organs, such as skin, liver, breast, and gastrointestinal (GI) tract.^3^, ^8^ Researchers have discovered that ANGPTL proteins’ transcriptional expression affected prognosis of patients in multiple types of cancer including lung cancer,^9^ colorectal cancer (CRC),^10^ liver cancer,^11^ etc However, the influences can be quite different across different types of ANGPTL proteins and cancers. As far as we know, only ANGPTL2^12^, ^13^ and ANGPTL4^14^, ^15^ have been discussed previously in GC patients. Therefore, we consider that it is necessary to investigate ANGPTL proteins’ transcriptional expression level across different clinicopathological situations and its relationship with prognosis of GC patients, thoroughly. In the present study, we implemented a deep bioinformatics analysis of ANGPTL proteins’ mRNA expression data together with available clinical data of GC patients based on several large public databases in order to illustrate their prognostic and potential therapeutic values in the treatment of GC.

## 2 MATERIALS AND METHODS

### 2.1 ONCOMINE data‐mining analysis

ONCOMINE (www.oncomine.org), an online web‐based cancer database of RNA and DNA sequences, was used to facilitate data mining the transcriptional expression level of ANGPTL proteins in 20 types of cancer.^16^ Transcriptional expression level of ANGPTL proteins in GC samples was compared with those in normal gastric samples using Student's *t*‐test. Statistically significant *P* value and fold change (FC) were demarcated as *P* < .05 and FC > 2, respectively.

### 2.2 TCGA‐STAD dataset

The Cancer Genome Atlas (TCGA) (https://cancergenome.nih.gov) contains gene expression data obtained by sequencing and has accurate clinicopathological data of many cancers. The stomach adenocarcinoma (STAD) dataset contains data from 375 GC tissues and 32 normal gastric tissues. The expression level of ANGPTL proteins was described using Log_2_(counts) together with 95% confidence interval (CI). Kruskal–Wallis (K–W) test was applied to compare expression level of different ANGPTL proteins. Further Dunn's multiple comparisons test would be conducted if the results of K–W test were of significance. *P* < .05 was considered to be significant. Transcriptional expression of ANGPTL proteins in GC samples was compared with those in normal gastric samples using R statistical software package (http://www.R‐project.org). *P* value < .01 and Log FC absolute value greater than 1 were considered as filter to find differentially expressed ANGPTL proteins.

### 2.3 LinkedOmics

The prognostic value of ANGPTL proteins’ mRNA transcription level was measured using an online portal, the LinkedOmics (www.linkedomics.org),^17^ which included gene expression profiles and clinical information of 375 GC patients from TCGA‐STAD. Patients with GC were separated into two groups based on median gene expression (high vs low). The overall survival (OS) of these two groups were compared by Cox regression analysis and demonstrated with Kaplan–Meier (K–M) survival curves. The hazard ratio (HR) and *P* value were calculated as well. *P* < .05 was considered significant.

### 2.4 Kaplan–Meier plotter

The prognostic value of ANGPTL proteins’ mRNA transcription level was also measured using an online open database, the Kaplan–Meier Plotter (www.kmplot.com),^18^ which included gene expression profiles and survival information of 876 GC patients from six other datasets rather than TCGA. Patients with GC were separated into two groups based on median gene expression (high vs low). The OS and post‐progression survival (PPS) of these two groups were compared by a Kaplan–Meier survival plot. The HR with 95% CI and Log‐rank *P* value were calculated. *P* < .05 was considered significant. We demonstrated K–M survival curves based on the value of each ANGPTL protein's most detected probe, and the number at risk was displayed below the curves.

### 2.5 cBioPortal

The cBioPortal (www. cbioportal.org) is an open‐access dataset for exploring multiple cancer genes. The GC dataset contains data from 415 cases, with pathologic diagnosis chosen by cBioPortal for further analyses of ANGPTL proteins.^19^, ^20^ The mRNA expression level of ANGPTL proteins in two groups of GC patients with different clinicopathological features was compared by Mann‐Whitney (M‐W) test. K‐W test was applied in multiple groups comparison. Further Dunn's multiple comparisons test would be conducted if the results of K‐W test were of significance. *P* < .05 was considered significant. Besides, genes with the highest expression correlation with each ANGPTL protein were generated by cBioPortal, and the top 120 co‐expressed genes with highest Spearman correlation score were included in the following functional enrichment analysis.

### 2.6 Metascape

Functional enrichment analysis was carried out using Metascape (http://metascape.org)^21^ and analyzed in context of gene ontology (GO)^22^ and Kyoto Encyclopedia of Genes and Genomes (KEGG)^23^ biological pathways. The genes were assigned to functional groups based on molecular functions, biological processes, and specific pathways.

## 3 RESULTS

### 3.1 Downregulated expression of ANGPTL1/3/6 in patients with GC

**FIGURE 1:**
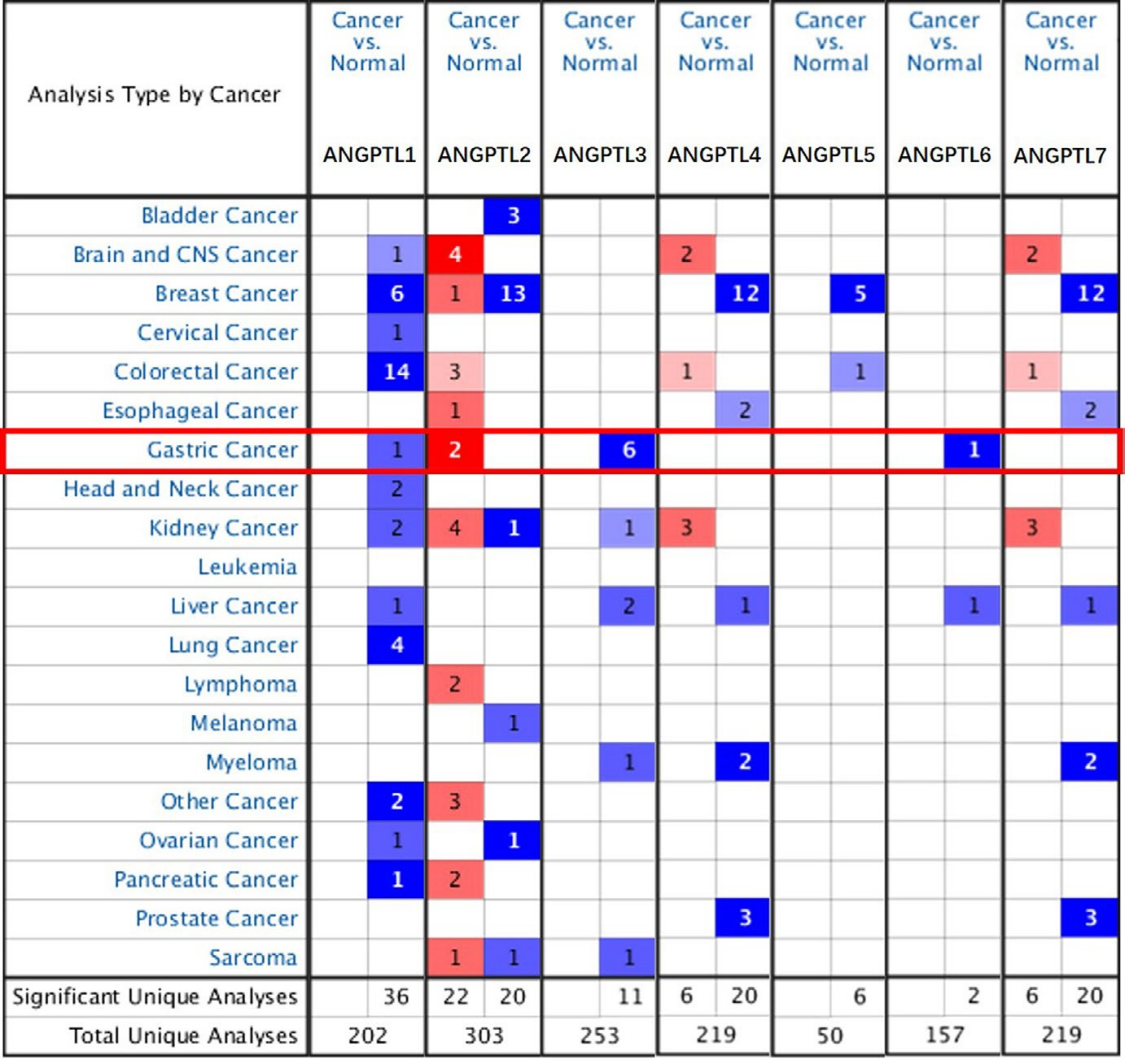
Significantly changed ANGPTL protein’s expression in different types of cancers. This information is attained from ONCOMINE and indicates the numbers of datasets with statistically significant (*P* value ≤ 10‐4, Fold Change ≥ 2 and Gene Rank ≥ Top 10%) mRNA high expression (Red) or low expression (Blue) of ANGPTL proteins (different types of cancer vs corresponding normal tissue). Cell color shade was decided by the best gene rank for the analyses within the cell, and the gene rank was analyzed by percentile of target genes in the top of all genes measured by each study

Seven ANGPTL proteins were identified using the ONCOMINE database,^16^ excluding ANGPTL8. As shown in Figure 1, we first measured the expression of ANGPTL proteins in 20 types of cancer samples and compared those to normal tissue samples. The mRNA expression of ANGPTL1/2/3/6 was significantly dysregulated in GC samples in multiple datasets.

In accordance with Table 1, ANGPTL1’s downregulation was observed in GC tissues compared with normal tissues, with a FC of −2.313 in Cui's dataset^24^ and a FC of −3.118 in DErrico's dataset,^25^ respectively. Overexpression of ANGPTL2 has been reported in diffuse gastric adenocarcinoma compared with normal gastric tissue, according to Chen^26^ (FC = 2.239) and DErrico's (FC = 2.360) dataset, while ANGPTL2 was also upregulated in gastric cancer (FC = 2.249) according to Wang's dataset.^27^ mRNA expression of ANGPTL3 was found to be downregulated in many types of GC compared to normal gastric tissues. Both Cho^28^ (FC = −2.092) and DErrico^25^ (FC = −3.247) exhibited elevating transcriptional level of ANGPTL3 in mixed‐type gastric adenocarcinoma. Markedly decreased expression of ANGPTL3 was reported in diffuse‐type GC and intestinal‐type GC, with FC = −2.090 and −3.599, by Cho and DErrico, respectively. In different datasets for ANGPTL3, we observed gastric adenocarcinoma having a FC = −2.074 compared with normal stomach reported by Cho,^28^ and a similar trend was found in Cui^24^ (FC = −3.703) and Wang's^27^ (FC = −4.953) datasets. Data from DErrico^25^ reported downregulated ANGPTL6 level in intestinal‐type gastric adenocarcinoma (FC = −3.797).

**TABLE 1:** Differential expression of ANGPTL proteins between different types of GC and normal gastric tissue. (ONCOMINE)

	Type	Fold change	*P* value	*t*‐test	REF
**ANGPTL1**	Gastric cancer	−2.313	1.22 × 10^−5^	−4.367	Cui
	Gastric cancer	−3.118	5.37 × 10^−4^	−3.568	DErrico
**ANGPTL2**	Gastric cancer	2.249	3.36 × 10^−5^	4.951	Wang
	Diffuse gastric adenocarcinoma	2.239	5.94 × 10^−5^	5.626	Chen
	Diffuse gastric adenocarcinoma	2.360	1.00 × 10^−3^	4.603	DErrico
**ANGPTL3**	Gastric adenocarcinoma	−2.074	3.00 × 10^−6^	−6.020	Cho
	Gastric mixed adenocarcinoma	−2.092	1.12 × 10^−6^	−6.607	Cho
	Diffuse gastric adenocarcinoma	−2.090	1.19 × 10^−6^	−6.659	Cho
	Gastric mixed adenocarcinoma	−3.247	1.26 × 10^‐5^	−5.714	DErrico
	Gastric intestinal‐type adenocarcinoma	−3.599	6.11 × 10^−7^	−5.459	DErrico
	Gastric cancer	−3.703	4.81 × 10^−6^	−4.574	Cui
	Gastric cancer	−4.953	1.00 × 10^−3^	−3.496	Wang
**ANGPTL6**	Gastric intestinal‐type adenocarcinoma	−3.797	1.68 × 10^−9^	−7.084	DErrico

GC, gastric cancer *t*‐test, Student's *t* test; REF, reference.

**FIGURE 2:**
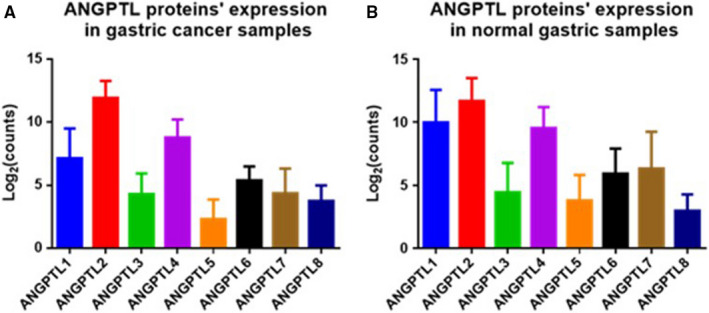
ANGPTL proteins’ expression level in TCGA‐STAD dataset. (A) ANGPTL proteins’ expression level in GC samples. (B) ANGPTL proteins’ expression level in normal gastric samples. GC: gastric cancer

**FIGURE 3:**
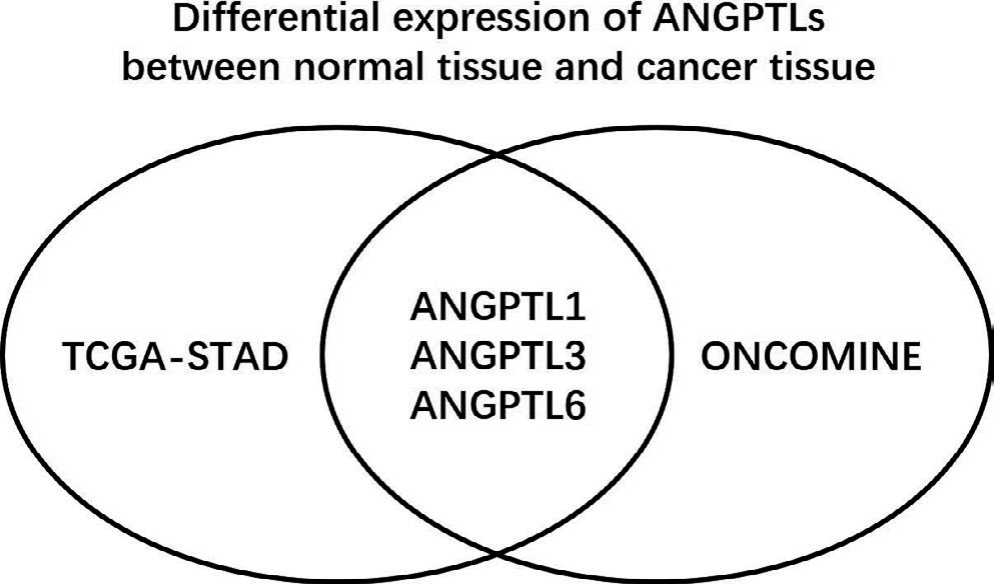
Differentially expressed ANGPTL proteins indicated by both TCGA‐STAD and ONCOMINE. GC: gastric cancer

Besides the datasets included by ONCOMINE, the transcriptional expression level of ANGPTL proteins in TCGA‐STAD dataset is also demonstrated in Table 2, Figure 2A and B. ANGPTL1/2/4 exhibited much higher expression level than other members of ANGPTL family no matter in gastric cancer samples or in normal gastric samples (Data not shown, all *P* values generated by Dunn's multiple comparisons test were less than .05). The mean expression level [measured by Log_2_(counts)] of ANGPTL1/2/4 was 9.982, 11.690, and 9.548 in normal tissue and 7.142, 11.930, and 8.792 in cancer tissue, respectively. When *P* value < .01 and Log FC absolute value greater than 1 were considered as filter, downregulated genes were ANGPTL1 (*P* = 4.480 × 10^−21^), ANGPTL3 (*P* = 4.140 × 10^−5^), ANGPTL4 (*P* = 3.882 × 10^−7^), ANGPTL5 (*P* = 1.490 × 10^−9^), ANGPTL6 (*P* = 4.150 × 10^−18^), and ANGPTL7 (*P* = 1.600 × 10^−25^). Therefore, both ONCOMINE database and TCGA‐STAD dataset indicated that ANGPTL1/3/6 were downregulated in gastric cancer samples (Figure 3).

**TABLE 2:** Differential expression of ANGPTL proteins between GC samples and normal gastric samples. (TCGA‐STAD)

Name	Log_2_ (counts) in normal sample	Log_2_ (counts) in cancer sample	Log FC	*P* Value	FDR
**ANGPTL1**	9.982 [9.049, 10.910]	7.142 [6.902, 7.382]	−2.772	**4.480** × **10** ^−^ **^21^**	2.210 × 10^−19^
ANGPTL2	11.690 [11.030, 12.340]	11.930 [11.790, 12.070]	−0.472	2.266 × 10^−2^	4.347 × 10^−2^
**ANGPTL3**	4.434 [3.579, 5.290]	4.294 [4.127,4.461]	−1.217	**4.140** × **10** ^−^ **^5^**	1.780 × 10^−4^
**ANGPTL4**	9.548 [8.950, 10.150]	8.792 [8.648,8.937]	−1.180	**3.882** × **10** ^−^ **^7^**	2.612 × 10^−6^
**ANGPTL5**	3.792 [2.990, 4.595]	2.307 [2.129,2.486]	−1.903	**1.490** × **10** ^−^ **^9^**	1.552 × 10^−8^
**ANGPTL6**	5.917 [5.187, 6.647]	5.386 [5.273, 5.498]	−1.729	**4.150** × **10** ^−^ **^18^**	1.610 × 10^−17^
**ANGPTL7**	6.324 [5.270, 7.379]	4.355 [4.154, 4.556]	−2.799	**1.600** × **10** ^−^ **^25^**	1.31 × 10^−23^
ANGPTL8	2.969 [2.484, 3.453]	3.744 [3.618, 3.870]	0.303	9.349 × 10^−2^	1.442 × 10^−1^

The bold text in first column highlights differentially expressed ANGPTL proteins (*P* < .01, absolute value of log FC > 1). The bold *P* values mean *P* < .01. FC, fold change; FDR, false discovery rate; GC, gastric cancer.

### 3.2 Upregulated ANGPTL1/2/4 were correlated with poor prognosis in GC patients

**FIGURE 4:**
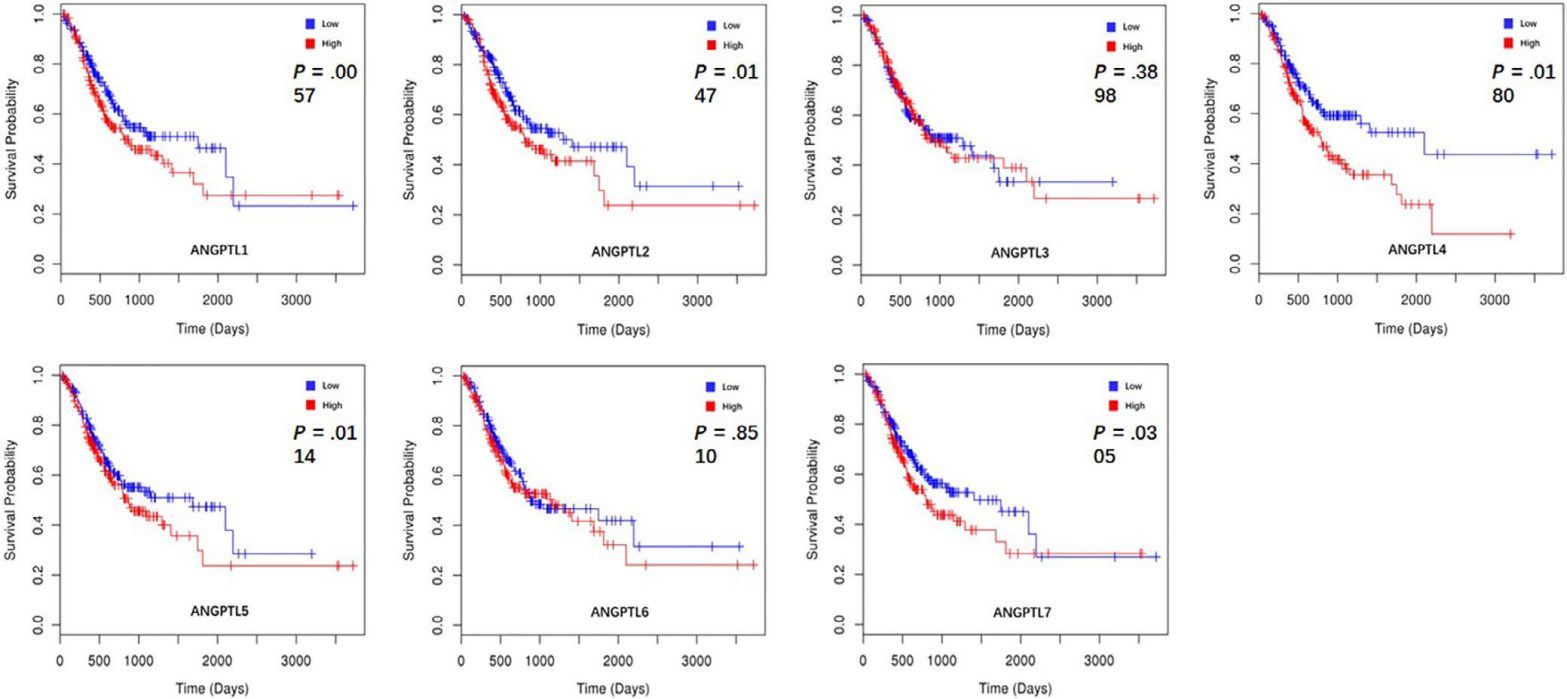
K–M curves revealed the association between OS and ANGPTL protein’s expression in GC patients (LinkedOmics). *P*: generated by Cox's regression test; Low: patients with ANGPTL protein's expression lower than median expression level; High: patients with ANGPTL protein's expression higher than median expression level; K–M: Kaplan–Meier; OS: overall survival; GC: gastric cancer

We separated all GC patients into two groups (high vs low) based on median expression values for each ANGPTL protein across all GC samples. As shown in Table 3 and Figure 4, seven ANGPTL proteins were identified using the LinkedOmics,^17^ excluding ANGPTL8. Cox regression test of survival data indicated that increased ANGPTL1 (≥5.119) [*P* = 5.731 × 10^−3^], ANGPTL2 (≥10.456) [*P* = 1.467 × 10^−2^], ANGPTL4 (≥7.062) [*P* = 1.799 × 10^−2^], ANGPTL5 (≥0.540) [*P* = 1.139 × 10^−2^], and ANGPTL7 (≥2.174) [*P* = 3.051 × 10^−2^] mRNA levels are associated with poor overall survival (OS) of GC.

**TABLE 3:** Cox regression revealed the association of ANGPTL proteins’ expression with prognosis in GC patients (LinkedOmics)

Name	Cutoff value [Log_2_ (RSEM)]	*P* value	FDR (BH)
**ANGPTL1**	5.119	**5.731 × 10^−3^**	1.375 × 10^−2^
**ANGPTL2**	10.456	**1.467 × 10^−2^**	4.401 × 10^−2^
ANGPTL3	1.342	3.898 × 10^−1^	5.847 × 10^−1^
**ANGPTL4**	7.062	**1.799 × 10^−2^**	1.248 × 10^−1^
**ANGPTL5**	0.540	**1.139 × 10^−2^**	1.367 × 10^−1^
ANGPTL6	3.421	8.510 × 10^−1^	8.510 × 10^−1^
**ANGPTL7**	2.174	**3.051 × 10^−2^**	5.525 × 10^−2^

The bold text in the first column highlights ANGPTL proteins with *P* < .05. The bold *P* values mean *P* < .05.

Cutoff value, median expression level; FDR (BH), FDR is calculated by BH (Benjamini‐Hochberg method); FDR, false discovery rate; GC, gastric cancer; *P* value, generated by Cox regression test.

**FIGURE 5:**
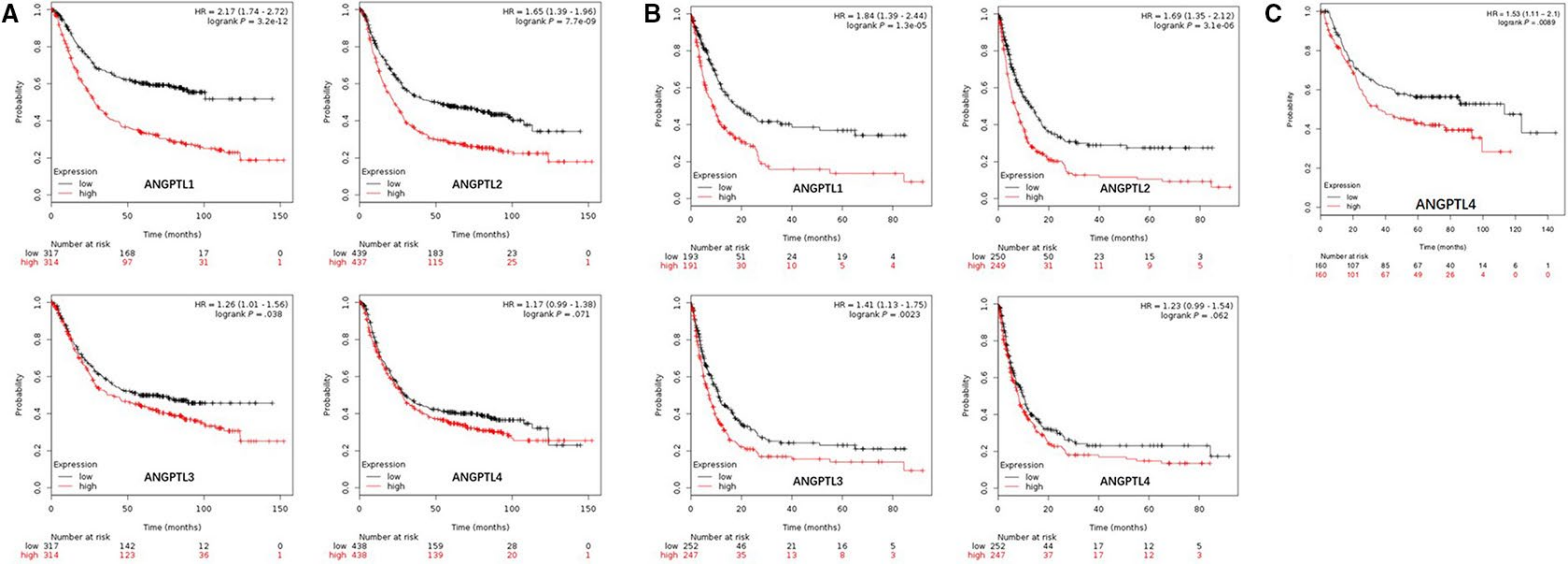
Upregulated ANGPTL1/2/4 were correlated with poor prognosis in GC patients according to K–M Plotter. (A) Kaplan‐Meier curves revealed the OS differences based on mRNA level of ANGPTL proteins in GC patients. (B) Kaplan‐Meier curves revealed the PPS differences based on mRNA level of ANGPTL proteins in GC patients. (C) Kaplan‐Meier curves revealed the OS differences based on mRNA level of ANGPTL4 in intestinal‐type GC patients. GC: gastric cancer; OS: overall survival; PPS: post‐progression survival; K–M Plotter: Kaplan–Meier plotter; HR: hazard ratio; Low: patients with ANGPTL protein's expression lower than median expression level; High: patients with ANGPTL protein's expression higher than median expression level

**FIGURE 6:**
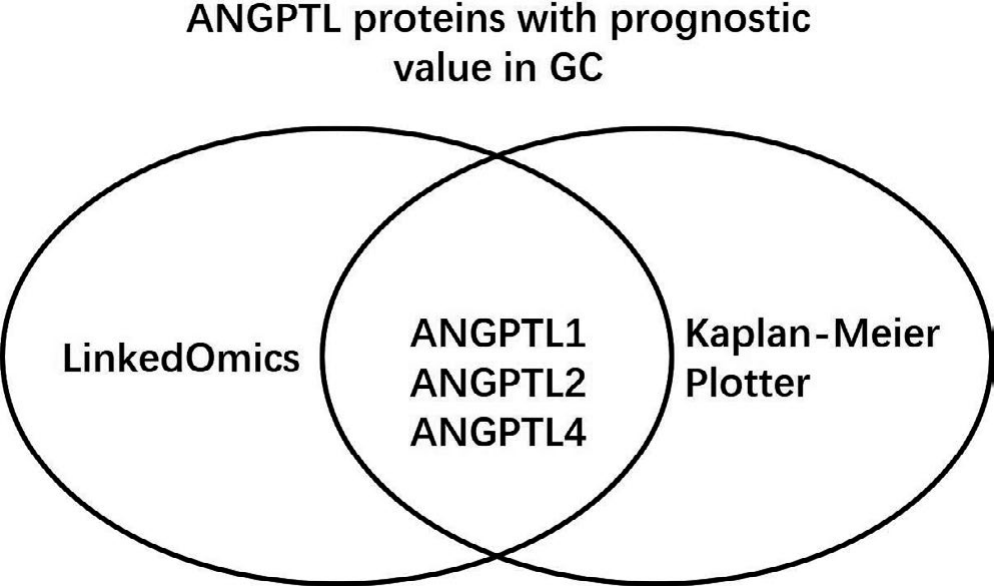
ANGPTL proteins with prognostic value in GC. High expression of ANGPTL1/2/4 predicted poor prognosis based on data from LinkedOmics and Kaplan‐Meier Plotter. GC: gastric cancer

We also investigated the prognostic value of ANGPTL proteins’ expression level using Kaplan–Meier Plotter^18^ and four members of the family could be identified. High expression of ANGPTL1 (≥27) predicted poor OS in 631 patients (HR: 2.17 [1.74, 2.72]; *P*: 3.2 × 10^−12^). Similar situations were observed in patients with ANGPTL2 counts ≥ 807 and ANGPTL3 counts ≥ 19, featuring a HR of 1.65 [1.39, 1.96] and a HR of 1.26 [1.01, 1.56], respectively. ANGPTL4 (≥100) predicted short OS (HR: 1.17 [0.99, 1.38]) with a marginal significance (*P* = 7.1 × 10^−2^) [Table 4(a) and Figure 5(A)]. In regard to ANGPTL1/2/3/4 expression's relationship with PPS, association similar to that with OS was found through K–M Plotter [Table 4(b) and Figure 5(B)]. Moreover, in 320 patients with intestinal‐type GC, upregulated ANGPTL4 (≥95) was related to poor OS with significance (HR: 1.53 [1.11, 2.10]; *P*: 8.9 × 10^−3^) [Figure 5(C)]. Therefore, we concluded that upregulated ANGPTL1/2/4 were correlated with poor prognosis in GC patients based on data from LinkedOmics and Kaplan–Meier Plotter [Figure 6].

**TABLE 4:** Association of ANGPTL proteins’ expression with prognosis in GC patients revealed by K–M Plotter

(a) Upregulated ANGPTL1/2/4 were correlated with short OS in GC patients					
Name	Cutoff value (counts)	Range (counts)	*P* value	HR	No. patients
**ANGPTL1**	27	1‐3573	**3.2** × **10** ^−^ **^12^**	2.17 [1.74, 2.72]	631
**ANGPTL2**	807	47‐6404	**7.7** × **10** ^−^ **^9^**	1.65 [1.39, 1.96]	876
**ANGPTL3**	19	0‐295	**3.8** × **10** ^−^ **^2^**	1.26 [1.01, 1.56]	631
**ANGPTL4**	100	2‐1564	**7.1** × **10** ^−^ **^2^**	1.17 [0.99, 1.38]	876
(b) Up‐regulated ANGPTL1/2/4 were correlated with short PPS in GC patients					
**ANGPTL1**	42	0‐3859	**1.3** × **10** ^−5^	1.84 [1.39, 2.44]	384
**ANGPTL2**	644	90‐6182	**3.1** × **10** ^−^ **^6^**	1.69 [1.35, 2.12]	499
**ANGPTL3**	22	1‐3085	**2.3** × **10** ^−3^	1.41 [1.13, 1.75]	499
**ANGPTL4**	95	5‐1564	**6.2** × **10** ^−2^	1.23 [0.99, 1.54]	499

The bold values in fourth column represent *P* values < .05.

Cutoff value, median expression level; GC, gastric cancer; HR, hazard ratio; K–M Plotter, Kaplan–Meier plotter; No. Patients, number of patients; *P* value, Log rank *P* value.

### 3.3 The association between ANGPTL1/2/3/4/6 expression level and clinicopathological features of GC patients

**FIGURE 7:**
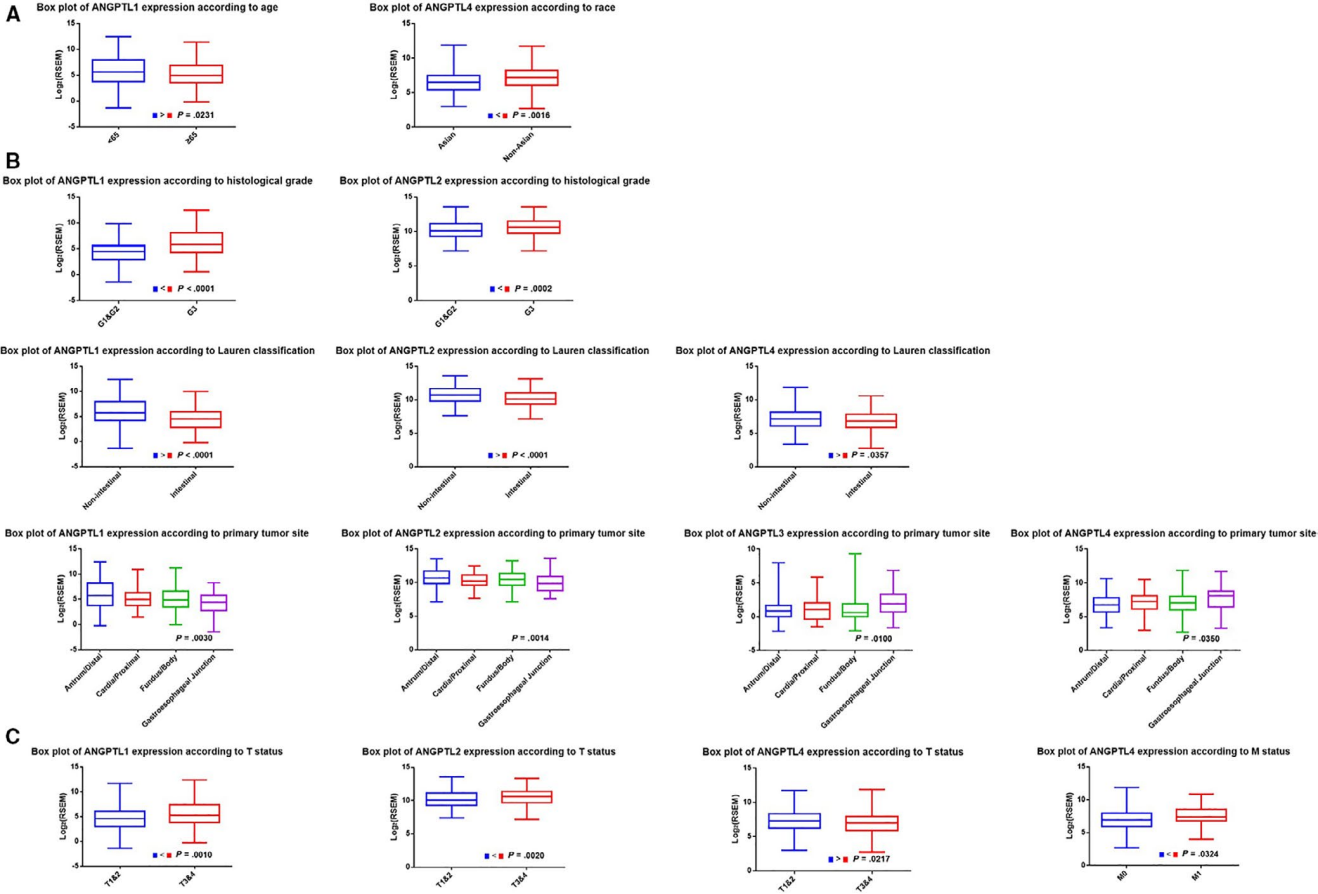
ANGPTL proteins with significantly changed expression according to various clinicopathological features among ANGPTL1/2/3/4/6 (cBioPortal). (A) Differently expressed ANGPTL proteins according to demographical features; (B) Differently expressed ANGPTL proteins according to pathological features; (C) Differently expressed ANGPTL proteins according to clinical staging. Clinical staging was based on the 7^t^h edition of AJCC TNM staging. Red boxes highlighted the box plots demonstrating significant difference between groups (*P* < .05). GC: gastric cancer

Taking ANGPTL proteins with aberrant expression or prognostic value into consideration, we next focused on the association between ANGPTL1/2/3/4/6 expression level and clinicopathological features of GC patients. M‐W test was implemented to compare the mRNA expression level of ANGPTLs between two groups of GC patients from cBioPortal^19^, ^20^ with different clinicopathological features. Only ANGPTL4 exhibited lower expression in Asian than in other races (*P* = .0016). For age criterion, there was no significant difference between <65 year and ≥65 year groups, except for ANGPTL1, which demonstrated reduced levels in the older group (*P* = .0231) (Figure 7A). No significant difference in ANGPTLs expression was observed across gender.

As shown in Figure 7(B), GC patients with higher histological grade (G3) showed higher expression of ANGPTL1 (*P* < .0001) and ANGPTL2 (*P* = .0002) than G1 and G2 patients. Intestinal‐type GC patients tended to express low levels of ANGPTL1 (*P* < .0001), ANGPTL2 (*P* < .0001), and ANGPTL4 (*P* = .0357) compared to patients with mixed‐ and diffuse‐type GC. The location of tumor lesion also affected the expression level of ANGPTL1 (*P* = .0030), ANGPTL2 (*P* = .0014), ANGPTL3 (*P* = .0100), and ANGPTL4 (*P* = .0350) according to K‐W test. Further Dunn's multiple comparisons test revealed increased ANGPTL1 (*P* = .0033)/2 (*P* = .0012) and decreased ANGPTL3 (*P* = .0071)/4 (*P* = .0030) expression in antrum/distal group compared to gastroesophageal junction group (Data not shown).

We found that the expression of ANGPTL1 (*P* = .0010) and ANGPTL2 (*P* = .0020) mRNA was significantly increased in T3 and T4 groups, while ANGPTL4 (*P* = .0217) mRNA expression was significantly reduced in T3 and T4 groups. Upregulated ANGPTL4 was also found to be associated with metastasis (*P* = .0324) (Figure 7C). Lymph node (N) status and clinical stage had no relationship with any of these ANGPTL protein’s expression level.

### 3.4 Functional enrichment analysis of genes co‐expressed with ANGPTL1/2/4

Besides differential expression in cancer tissue and prognostic value, ANGPTL proteins’ expression level in gastric tissue was also taken into account when we selected candidates for functional enrichment analysis. Since ANGPTL1/2/4 exhibited much higher expression level than other members of ANGPTL family in gastric tissue, we decided to further investigate their roles in GC genesis and development. The top 120 genes that had the most significant correlation with ANGPTL1/2/4 were generated by cBioPortal^19^, ^20^ and were included in the following functional enrichment analysis using Metascape.^21^

**FIGURE 8:**
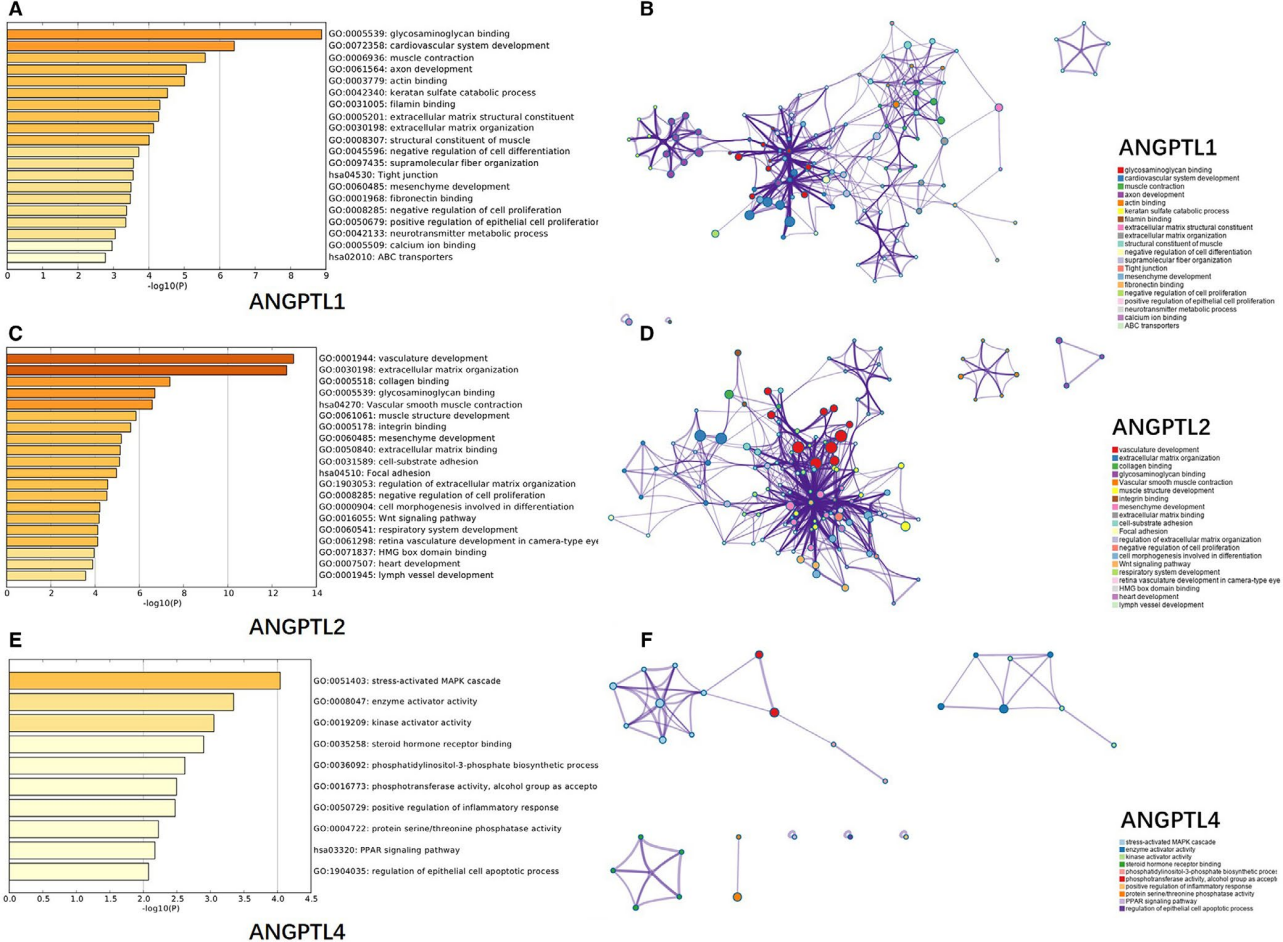
Functional enrichment analysis of genes co‐expressed with ANGPTL1/2/4 using Metascape. (A), (C), (E): Heatmaps of the molecular functions, biological processes, or pathways enriched with ANGPTL1/2/4 co‐expressed genes. The bar color shade was decided by the *P* value, the deeper the shade, the less the *P* value. (B), (D), (F): Networks exhibiting interactions among the clusters of genes enriched in the molecular functions, biological processes, or pathways presented in the heatmaps (A), (C), (E), respectively. The points in different colors represented clusters of genes enriched in different molecular functions, biological processes, and pathways. The purple lines between points represented interactions between genes, the shorter the line, the closer the relationship. ABC transporters: ATP‐binding cassette transporters; HMG box: high mobility group box; MAPK cascade: mitogen‐activated protein kinase cascade; PPAR signaling pathway: peroxisome proliferator‐activated receptor

As shown in Figure 8(A), the top 120 genes co‐expressed with ANGPTL1 were mainly enriched in molecular functions, biological processes, and pathways involved in interactions with extracellular matrix (ECM) (eg, glycosaminoglycan binding, keratan sulfate catabolic process, and ECM structural constituent and organization), cell differentiation and proliferation (eg, positive regulation of epithelial cell proliferation), tissue development (eg, mesenchyme development), and muscle system (eg, actin binding). Figure 8(B) was a network that exhibited the interactions among cluster of genes enriched in the molecular functions, biological processes, and pathways mentioned above. We could see that the genes enriched in ECM‐related functions showed closer relationship with those enriched in cell differentiation and proliferation and tissue development‐related processes.

According to Figure 8(C), ANGPTL2 probably participate in the development of tumor microenvironment, especially vasculature development. It may also regulate ECM binding and adhesion. Meanwhile, ANGPTL2 could possibly pose influences on cell proliferation and differentiation. Wnt signaling pathway and high mobility group box (HMG box) domain binding were among the top 20 functions enriched with ANGPTL2 co‐expressed genes, too. Figure 8(D) illustrated an intimate relationship between the genes enriched in ECM organization‐ and adhesion‐related functions and those enriched in vasculature development‐related processes.

The genes co‐expressed with ANGPTL4 did not enrich in some specific biological processes as remarkable as ANGPTL1/2 did. However, ANGPTL4 seemed to play a role in multiple different processes rather than only angiogenesis‐related ones. It probably took part in the stress‐activated mitogen‐activated protein kinase cascade (MAPK cascade), peroxisome proliferator‐activated receptor (PPAR) signaling pathway, phosphatidylinositol‐3‐phosphate (PI3P) biosynthetic process, epithelial cell apoptotic process, and regulation of inflammatory response. ANGPTL4 may also associate with activity as enzyme/kinase activator, phosphotransferase/phosphatase, or steroid hormone receptor binding process (Figure 8(E)). The network in Figure 8(F) revealed the association between genes enriched in stress‐activated MAPK cascade and those enriched in PI3P biosynthetic process, phosphotransferase activity function. Genes enriched in enzyme/kinase activator activity function only interacted with each other.

## 4 DISCUSSION

ANGPTL proteins are a family of secreted glycoproteins which participate in multiple biological processes, mainly including angiogenesis, inflammation, and metabolism.^3^ During the last decade, emerging evidences revealed ANGPTL proteins’ roles in regulating different steps of carcinogenesis and metastasis through their effects on the processes mentioned above. Recently, Carbone et al^3^ published a systematic review outlining the current knowledge about ANGPTL proteins’ functions in angiogenesis, inflammation, cancer progression, and metastasis. They also discussed the most recent evidences sustaining ANGPTL proteins’ role as prognostic biomarkers for cancer therapy. However, the roles of ANGPTL proteins in cancer progression and metastasis can be quite tumor type‐dependent, even researches investigating the same type of cancer generate conflicting conclusions sometimes. So far, few articles have explored the mRNA expression of ANGPTL proteins in GC.^12^, ^14^ Their prognostic value and biological functions in GC remain to be elucidated, too. To our knowledge, this is the first study to exhibit ANGPTL proteins’ transcriptional expression in GC comprehensively and to identify specific ANGPTL proteins with prognostic value, and biological functions in GC development using integrative bioinformatics.

### 4.1 ANGPTL proteins’ mRNA expression in gastric tissues

Carbone et al have briefly summarized ANGPTL family members’ mRNA expression level in esophageal and colorectal tissues based on previous published researches. ANGPTL1 was highly expressed in all parts of the GI tract except esophagus.^3^ ANGPTL2 exhibited high expression in esophageal^3^ and colorectal cancer.^10^ The expression level of ANGPTL4/6/7 was also high in CRC.^29^, ^30^, ^31^ Our article revealed significantly higher expression level of ANGPTL1/2/4 than that of ANGPTL3/5/6/7 in gastric tissues through bioinformatics, which was not demonstrated in Carbone's review.^3^ The information mentioned above together may provide us a rough impression of ANGPTL proteins’ mRNA expression level landscape along the GI tract.

### 4.2 ANGPTL1: expression, prognostic value, and roles in GC

According to our results, both ONCOMINE database and TCGA‐STAD dataset showed that ANGPTL1 were downregulated in GC samples compared to normal gastric samples and prompted ANGPTL1’s potential inhibitory role in gastric tumorigenesis. Downregulation of ANGPTL1 was observed in kidney, lung, prostate, bladder, and thyroid cancers, too.^3^ In fact, ANGPTL1 was generally supported to be a tumor suppressor across different types of tumor by both in vitro and in vivo experiments. For example, ANGPTL1 overexpression in breast cancer (BC) cells could result in a significant reduction in the number and size of tumor nodules.^32^ Primary melanoma tumors derived from ANGPTL1‐secreting cells grew more slowly in vivo compared to empty vector‐transfected cells.^32^ It has also been reported that ANGPTL1 treatment remarkably inhibited in vitro and in vivo migration and invasion ability of hepatocellular carcinoma (HCC) cells.^11^ Meanwhile, immunohistochemistry (IHC) analysis of HCC samples revealed that patients with higher level of ANGPTL1 expression had less metastasis as well as longer survival time.^11^

Several possible mechanisms underlie this suppressive activity. Firstly, ANGPTL1 may play an essential role in tumor inhibition by balancing angiogenesis and permeability. It could both inhibit VEGF‐induced endothelial cells proliferation and induce extracellular signal‐regulated kinase 1/2 (ERK1/2)‐related antiapoptotic activity..^32^ Secondly, ANGPTL1 was responsible for reorganization of cytoskeleton through inhibition of actin stress fiber formation, which probably result in an altered cellular morphology. Thirdly, ANGPTL1 could induce mesenchymal‐to‐epithelial transition (MET) through integrin α1β1, miR‐630, and SLUG (SNAIL‐related zinc‐finger transcription factor) pathway, thus allow cancer cells to regain epithelial properties.^9^

However, according to our results, upregulated ANGPTL1 was correlated with poor prognosis, higher histological grade, non‐intestinal Lauren classification, and advanced T stage in GC patients suggesting a GC‐promoting role of this molecule. Perhaps, ANGPTL1 played different roles in gastric tumorigenesis and gastric tumor progression. There was a lack of researches focusing on ANGPTL1’s roles in GC. Our functional enrichment analysis indicated several pathways by which ANGPTL1 exerted influence on GC progression.

Most of the pathways are related to interactions with ECM (eg, glycosaminoglycan binding, keratan sulfate catabolic process, and ECM structural constituent and organization), cell differentiation and proliferation (eg, positive regulation of epithelial cell proliferation), tissue development (eg, mesenchyme development), and muscle system (eg, actin binding). Further studies could be conducted based on this analysis.

### 4.3 ANGPTL2: expression, prognostic value, and roles in GC

ANGPTL2 has been proved to be tumor‐promoting among several types of cancer. High ANGPTL2 expression level has been observed in many types of cancer including esophageal,^3^ colorectal,^10^ prostate,^5^ pancreatic,^33^ lung,^34^ breast,^35^ liver,^36^ and skin^37^ cancers. ANGPTL2 mainly exerted its pro‐angiogenic and antiapoptotic abilities in the tumor microenvironment. ANGPTL2 also increased cancer cells’ migratory and invasive ability, thus facilitate tumor metastases through different mechanisms. For instance, an autocrine signaling between ANGPTL2 and its receptor LILRB2 was able to induce early EMT and tumor progression in preneoplastic pancreatic ductal cells.^33^ ANGPTL2 also strengthened responsiveness of BC cells to chemokine (C‐X‐C motif) ligand 12 (CXCL12) by the upregulation of C‐X‐C motif receptor 4 (CXCR4), thus promoted these cells’ recruitment to bone metastatic sites.^35^ In addition, ANGPTL2 induced inflammation and oxidative stress, generating a tumor microenvironment that supported methylation, and consequently reducing gene expression of DNA repair enzymes, such as mutS homolog 2 (MSH2), leading to DNA mutations and cancer initiation in an experimental model of skin cancer.^37^

Our bioinformatics analysis revealed elevated expression of ANGPTL2 in GC tissue compared to normal gastric tissue based on data from ONCOMINE and no significant difference in ANGPTL2 expression between normal and cancer tissue based on data from TCGA‐STAD. Besides, upregulated ANGPTL2 was also correlated with poor prognosis, higher histological grade, non‐intestinal Lauren classification, and advanced T stage in GC patients according to our results. Therefore, we may conclude that ANGPTL2 behave as a tumor promoter in GC just like in many other types of cancer. Several studies on ANGPTL2 and GC generated similar conclusion as well. As in vitro researches indicated, ANGPTL2 knockdown caused anoikis and inhibited proliferation, invasion, and migration in GC cells,^12^ while proliferation rate and invasive ability in ANGPTL2‐overexpressed GC cells were higher than in control cells.^13^ Besides, higher expression of ANGPTL2 was observed in highly malignant and undifferentiated GC cell lines.^13^, ^38^ Clinical researches prompted that upregulated ANGPTL2 was associated with GC progression, early recurrence, and poor prognosis.^12^ Moreover, ANGPTL2 could be a potential novel noninvasive biomarker for GC. The serum ANGPTL2 level of GC patients were significantly higher than those of healthy controls. Recent studies reported receiver operating characteristic (ROC) curves which yielded robust AUC value (0.831) accompanied by high sensitivity (73.0%) and specificity (82.2%) in distinguishing GC patients from healthy controls.^12^

Although ANGPTL2’s GC promotive activity was observed by both in vitro studies and clinical researches, few studies explored the possible underlying mechanisms. According to our functional analysis of co‐expressed genes in GC, ANGPTL2 probably participate in the development of tumor microenvironment, especially vasculature development. It may also regulate ECM binding and adhesion. Meanwhile, ANGPTL2 could possibly pose influences on cell proliferation and differentiation. Wnt signaling pathway and HMG box domain binding were among the top 20 functions enriched with ANGPTL2 co‐expressed genes, too. These candidate pathways mentioned above needed further validation of experiments and could be hints of future mechanism studies.

### 4.4 ANGPTL3: expression and prognostic value in GC

Few articles about ANGPTL3’s role in cancer growth and invasion were reported. Existing studies showed contradicting results in different types of cancer. For instance, ANGPTL3 was significantly upregulated in oral squamous cell carcinoma (OSCC)‐derived cell lines compared to normal tissues. In vitro and in vivo OSCC models showed that ANGPTL3 knockdown arrested cell cycle at G1 phase through upregulating cyclin‐dependent kinase inhibitors, thus reduced cancer cell proliferation and growth^39^ Nonetheless, in HCC cells, ANGPTL3 inhibited cell proliferation and invasion through downregulation of p38MAPK and MMP‐9 cascade's activation.^40^

ANGPTL3 exhibited low expression in gastric tissues, though ANGPTL3 was downregulated in GC tissues compared to normal gastric tissues suggesting ANGPTL3’s role as a GC suppressor. However, the difference in expression level did not make a difference in GC patients’ prognosis and was not associated with other clinicopathological factors, except primary tumor site. Therefore, we are prone to the viewpoint that ANGPTL3 may not play an important role in regulating GC genesis and progression.

### 4.5 ANGPTL4: expression, prognostic value, and roles in GC

Recent researches revealed ANGPTL4’s wide‐spectrum of action including cancer growth, angiogenesis, metabolism, and metastasis. However, it seemed that ANGPTL4 acted in a tumor type‐dependent manner and even the findings in the same type of cancer contradicted with each other sometimes, too.

For instance, ANGPTL4 could be induced by hypoxia through upregulation of PGE2 receptor in CRC, thus promoted cancer cell proliferation. It could also stimulate a redox‐based mechanism which enhanced tumor cell survival by alteration of the O_2_ to H_2_O_2_ ratio and led to the activation of extracellular signal‐regulated kinase in CRC.^14^ Inconsistent with upregulated expression in CRC, ANGPTL4 expression was significantly lower in HCC tissues than in nontumor tissues. Low expression of ANGPTL4 was significantly associated with advanced tumor stage, poor differentiation as well as poor overall and disease‐free survival (DFS) of HCC patients.^15^ However, a study showed that serum ANGPTL4 protein is higher in HCC patients than in normal controls.^41^ Besides, ANGPTL4 was reported to be expressed at higher level in the blood of BC patients^42^ and high expression of ANGPTL4 correlated with a minor DFS of young BC patients.^43^ However, an in vitro study indicated that PPAR β/δ‐regulated ANGPTL4 strongly inhibited the transforming growth factor β (TGF β)‐induced invasion of MDA‐MB‐231 human BC cells.^44^

ANGPTL4’s proteolytic cleavage generated two isoforms of itself: an N‐terminal coiled‐coil domain (nANGPTL4), and a large fibrinogen‐like COOH‐terminal domain (cANGPTL4). Whereas the former was mainly involved in the endocrine regulatory role of lipid metabolism, insulin sensitivity, and glucose homeostasis, the latter may be a key regulator of the complex signaling during cancer development.^3^ The complexity of ANGPTL4’s role in cancer genesis and development probably result from the alteration of cleavage and posttranslational modification.^45^

Our integrated bioinformatics analysis further demonstrated the complexity of ANGPTL4’s role in gastric cancer. According to data from TCGA‐STAD, ANGPTL4 exhibited lower expression in GC tissue than in normal gastric tissue and higher expression in early T stage than advanced T stage. However, elevated ANGPTL4 expression level was also correlated with poor prognosis, unfavorable Lauren classification, and metastasis in GC patients. Previous researches focusing on ANGPTL4 and GC could not provide consistent conclusion, neither. Kubo et al suggested that hypoxia‐induced ANGPTL4 expression is independent of hypoxia‐inducible factor‐1α (HIF‐1α) in hypoxic GC cells and ANGPTL4 may be a favorable marker for predicting a long survival time.^46^ Meanwhile, Baba et al demonstrated that hypoxia‐induced ANGPTL4 expression was regulated by HIF‐1α in scirrhous GC cells and was essential for tumor growth, metastasis, and resistance to anoikis through different mechanisms, including downregulation of c‐Myc and focal adhesion kinase (FAK)/Src/phosphoinositide 3‐kinase (PI3K)‐protein kinase B (Akt)/ERK pathway, upregulation of p27, and apoptotic factors caspases‐3, −8, and −9.^47^ Besides, Tan et al found that cANGPTL4 bearing T266M mutation (T266M cANGPTL4) bound to integrin α5β1 with a reduced affinity compared to wild‐type cANGPTL4, leading to weaker activation of downstream signaling molecules. The tumors with T266M cANGPTL4 exhibited impaired proliferation, anoikis resistance, migratory capability, and had reduced adenylate energy charge. Further investigations also revealed that cANGPTL4 regulated the expression of glucose transporter 2 (Glut2).^48^

Many of the mentioned pathways by which ANGPTL4 exerted influence on other types of cancer were enriched in our functional enrichment analysis of ANGPTL4 co‐expressed genes in GC, such as stress‐activated MAPK cascade, PPAR signaling pathway, PI3P biosynthetic process, epithelial cell apoptotic process, and regulation of inflammatory response. However, some of them have not been verified by experiments in GC, perhaps further researches are needed.

### 4.6 ANGPTL6: expression and prognostic value in GC

Similar to ANGPTL3, ANGPTL6 also showed low expression in gastric tissues and was downregulated in GC tissues. Based on our results, ANGPTL6’s expression level did not exhibit prognostic value in GC patients and was not associated with other clinicopathological factors, neither. All the information seemed to suggest that ANGPTL6 may not exert much influence on GC genesis and progression.

However, it has been reported that the interaction between hepatic ANGPTL6 and tumoral integrin/E‐cadherin drives liver homing and colonization by CRC cells. Furthermore, an angiopoietin‐like 6‐mimicking peptide was capable of interfering with this interaction, thus acting as an antimetastatic compound.^30^ Hence, we guessed that it was still a possible research direction to investigate ANGPTL6’s role in liver metastasis of GC.

### 4.7 ANGPTL1/2/4 and resistance of antiangiogenesis agents

Emerging evidences has already demonstrated ANGPTL proteins’ potential roles in resistance of antiangiogenesis agents in other types of cancer. For example, a study indicated that ANGPTL1 could inhibit sorafenib resistance and cancer stemness in HCC cells through acting as a Met receptor inhibitor.^49^ Besides, ANGPTL2 was proved to be among the pro‐inflammatory factors overexpressed in pancreatic cancer (PC) cells which led to epithelial‐to‐mesenchymal transition (EMT) and resistance of anti‐VEGF treatment.^50^, ^51^ Moreover, in a research on triple‐negative breast cancer (TNBC), heparin‐binding epidermal growth factor (HB‐EGF) was found to play a pivotal role in the acquisition of tumor aggressiveness by regulating both ANGPTL4 and VEGFA.^52^ We thought this research indicated that only blocking VEGFR may not be enough to shut down angiogenesis in TNBC. Whether the ANGPTL proteins participated in the mechanism of antiangiogenesis drug resistance in GC or not has not been studied thoroughly before.

## 5 CONCLUSIONS

In the current study, we systematically analyzed transcriptional expression level and prognostic value of ANGPTL proteins in GC patients. We also exhibited the association of expression level with clinicopathological features and supplied a functional enrichment analysis. Integrative bioinformatics analysis suggests that ANGPTL1/2/4, compared to other ANGPTL proteins, may be potential therapeutic targets in GC patients. Among ANGPTL1/2/4, ANGPTL2 tends to be a GC promoter according to our results. However, we cannot conclude whether ANGPTL1/4 are GC promoter or suppressor based on the diverse information provided by our analysis. The ANGPTL proteins’ roles in GC are so complex that more well‐conducted clinical researches and in‐depth experiments are required to validate the diagnostic value of these ANGPTL proteins and explore the underlying mechanism by which ANGPTL proteins influence GC’s development.

## CONFLICT OF INTEREST

The authors declare that they have no competing interests.

## AUTHOR CONTRIBUTIONS

CT and EC conceived the project; CT, EC, HW, and KP analyzed the data, CT, XC, and YW drafted the manuscript; TL and YC had critically read the manuscript; SY and YY edited and reviewed the manuscript. All authors read and approved the final manuscript.

## ETHICS APPROVAL AND CONSENT TO PARTICIPATE

Not applicable.

## Supporting information

Fig S1

Fig S2

Fig S3

File S1

File S2

## Data Availability

The data utilized in this study are publicly available and listed below: ONCOMINE (www.oncomine.org); TCGA (https://cancergenome.nih.gov); LinkedOmics (www.linkedomics.org); Kaplan–Meier Plotter (www.kmplot.com); cBioPortal (www. cbioportal.org); Metascape (http://metascape.org).
