# Supplementary figures and images for: Mining the role of angiopoietin‐like protein family in gastric cancer and seeking potential therapeutic targets by integrative bioinformatics analysis
Source: Cancer Med. 2020 May 14;9(13):4850–63. doi: 10.1002/cam4.3100 (PMC7333835; doi:10.1002/cam4.3100)

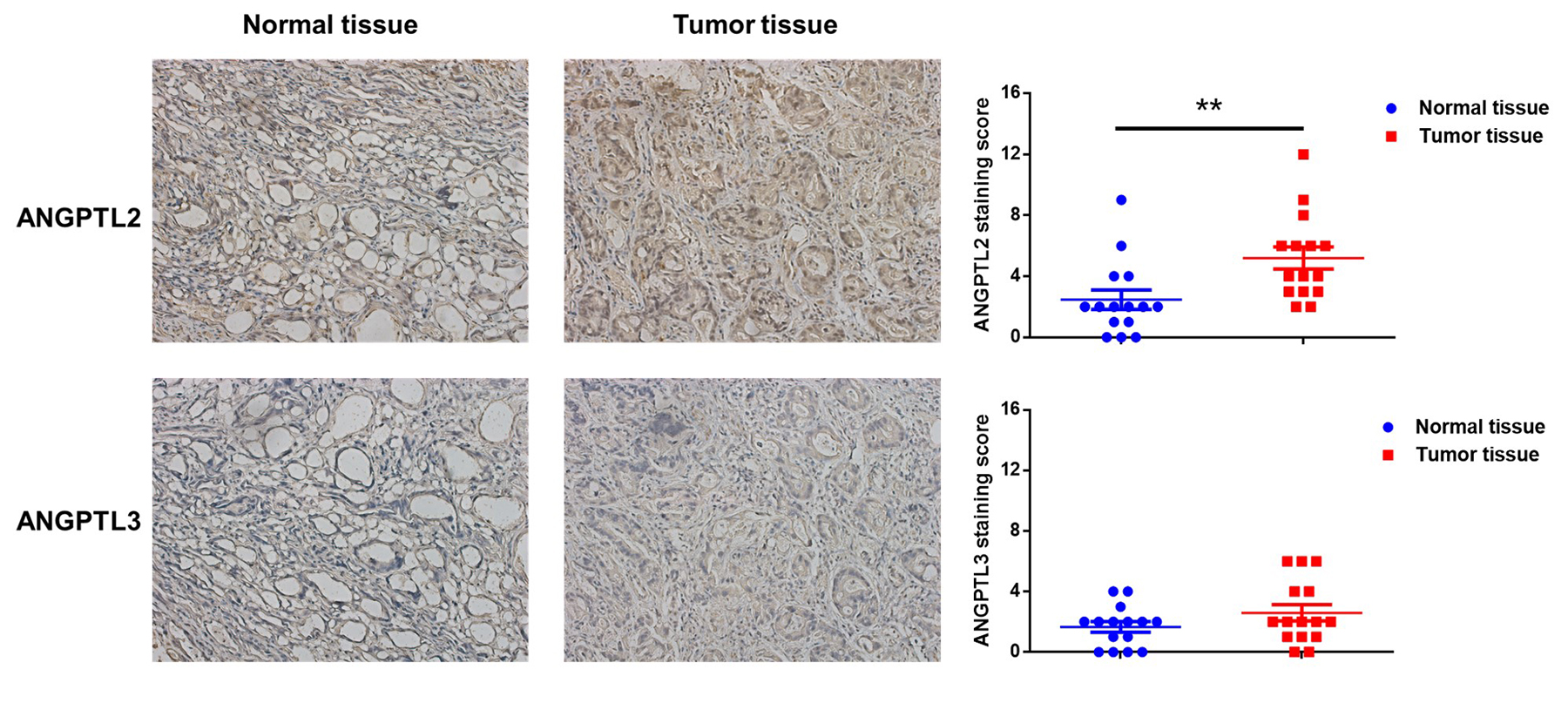

Supplement: Supplementary file 1 — Fig S1 [file CAM4-9-4850-s001.jpg]

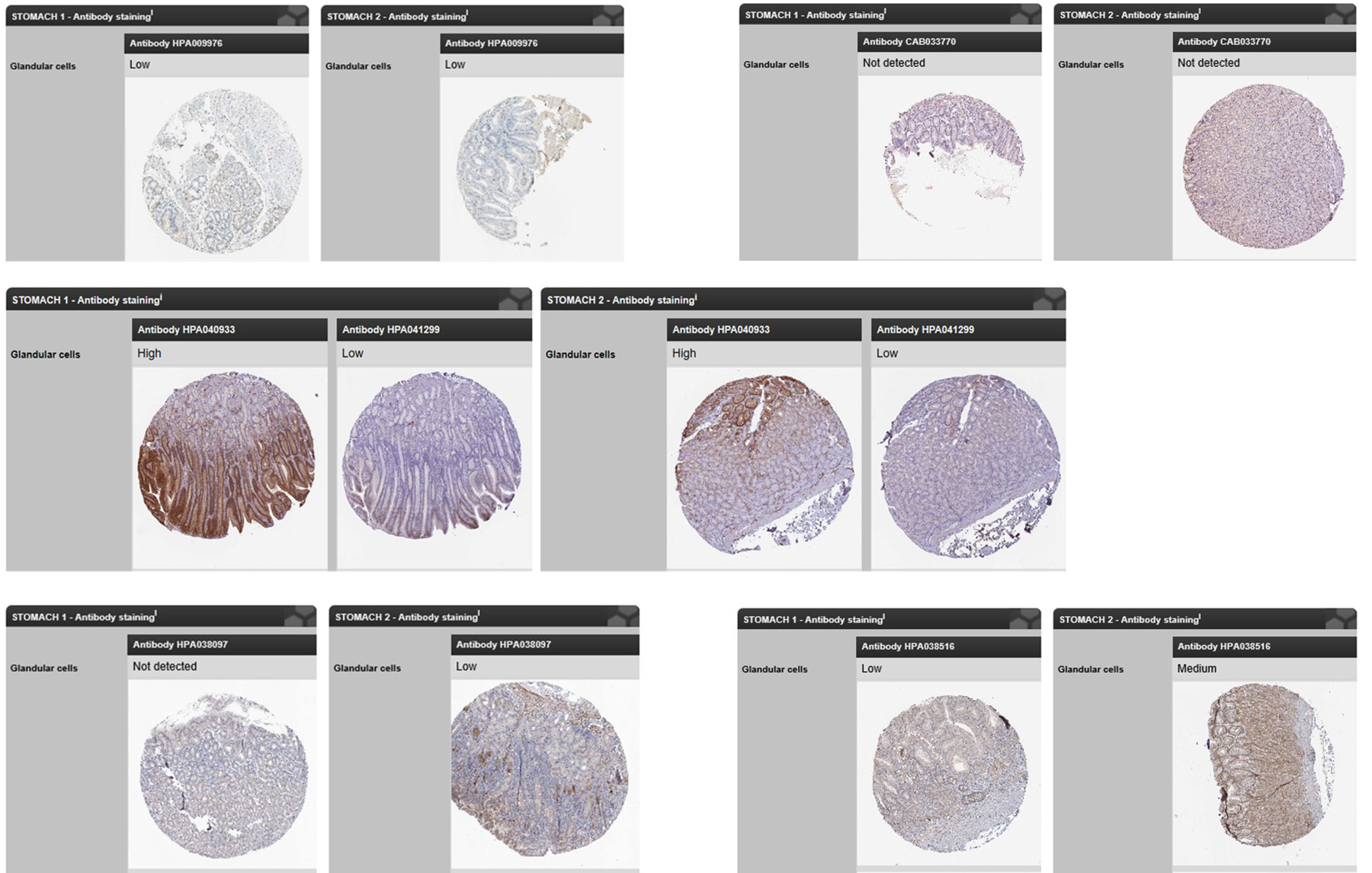

Supplement: Supplementary file 2 — Fig S2 [file CAM4-9-4850-s002.jpg]

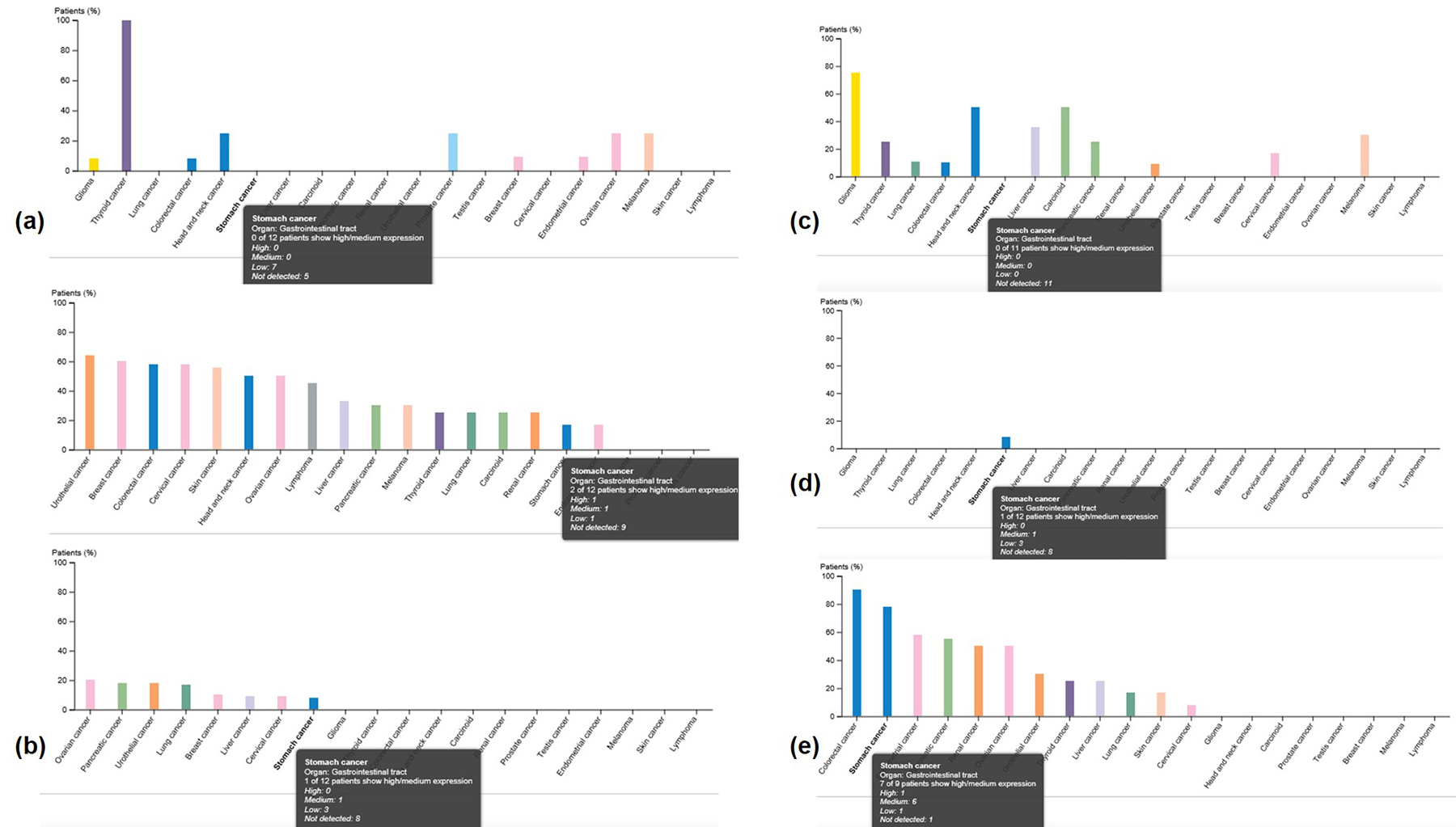

Supplement: Supplementary file 3 — Fig S3 [file CAM4-9-4850-s003.jpg]
