# Supplementary material for: Mining the role of angiopoietin‐like protein family in gastric cancer and seeking potential therapeutic targets by integrative bioinformatics analysis
Source: Cancer Med. 2020 May 14;9(13):4850–63. doi: 10.1002/cam4.3100 (PMC7333835; doi:10.1002/cam4.3100)
Supplement: Supplementary file 4 — File S1 [file CAM4-9-4850-s004.docx]

**Materials and methods used in supporting information**

**Tissue samples collection**

15 normal gastric tissue samples and 15 gastric cancer tissue samples were collected from March to June in 2019 at Zhongshan Hospital, Fudan University. The normal gastric tissue samples were obtained from healthy volunteers undergoing endoscopy. The gastric cancer tissue samples were obtained from pathologically diagnosed gastric cancer patients undergoing radical resection. Informed consent was obtained from all of the patients, and the Zhongshan Hospital ethics committee approved the research protocols for this study.

**Immunohistochemistry (IHC)**

The 30 tissue samples fixed by formalin were used for immunohistochemistry (IHC). The IHC of each sample was performed with rabbit anti‐ANGPTL2 antibody and anti-ANGPTL3 antibody (proteintech; dilution 1:200), respectively. The staining intensity was graded as 0 (absent staining), 1 (weak staining), 2 (moderate staining), and 3 (strong staining). The percentage of the staining was divided into five levels: no positive cells were recorded as 0, less than 25% positive cells were recorded as 1, 25%‐50% positive cells were recorded as 2, 50%‐75% positive cells were recorded as 3, and more than 75% positive cells were recorded as 4. The final staining score for each sample was calculated by multiplication, and the value range was between 0 and 12.

**Statistical methods**

The final staining score of ANGPTL 2/3 in GC and normal gastric tissue were compared by Mann-Whitney (M-W) test. P<0.05 was considered significant.

**Proteinatlas**

Proteinatlas (http://www.proteinatlas.org/), an online web-based database of proteins’ expression in different types of tissue (both normal and cancer tissues), was used to figure out the IHC staining of ANGPTL protein family in normal gastric tissue samples and gastric cancer tissue samples.
