# Supplementary material for: Mining the role of angiopoietin‐like protein family in gastric cancer and seeking potential therapeutic targets by integrative bioinformatics analysis
Source: Cancer Med. 2020 May 14;9(13):4850–63. doi: 10.1002/cam4.3100 (PMC7333835; doi:10.1002/cam4.3100)
Supplement: Supplementary file 5 — File S2 [file CAM4-9-4850-s005.docx]

**Results in supporting information**

**IHC results of ANGPTL2/3 obtained from 30 clinical tissue samples**

According to our IHC results, ANGPTL2 was over expressed in GC (P=0.002)，while ANGPTL3 showed no differential expression (P=0.302).**[Supplementary Figure 1]**

**IHC results of ANGPTL protein family obtained from Proteinatlas**

The expression level of ANGPTL1 was low in 2 normal gastric tissues, and among the 12 GC samples, 7 showed low ANGPTL1 expression, the other 5 showed no ANGPTL1 expression **[Supplementary Figure 2(a)&3(a)]**. Proteinatlas conducted IHC staining of two different ANGPTL2 antibodies (HPA040933 & HPA041299) in normal tissues and GC tissues. The former’s expression level was high in 2 normal gastric tissues, and among the 12 GC samples, 2 showed high to medium expression. Meanwhile, the latter’s expression level was low in 2 normal gastric tissues, and among the 12 GC samples, 1 showed medium expression. **[Supplementary Figure 2(b)&3(b)]** According to the IHC staining, we found that ANGPTL3 was not detected in all 11 GC tissues, and only showed low expression in one normal tissue sample. **[Supplementary Figure 2(c)&3(c)]** We also found that ANGPTL4 was not detected in 2 normal tissues, and showed medium (n=1) to low (n=3) expression in 12 GC samples. **[Supplementary Figure 2(d)&3(d)]** Finally, ANGPTL5 exhibited low to medium expression level in normal tissues and 7 of 9 GC samples exhibited medium (n=6) to high (n=1) expression. **[Supplementary Figure 2(e)&3(e)]**
